# Selective Migration of Subpopulations of Bone Marrow Cells along an SDF-1*α* and ATP Gradient

**DOI:** 10.1155/2014/182645

**Published:** 2014-12-31

**Authors:** Michael Laupheimer, Anna Skorska, Jana Große, Gudrun Tiedemann, Gustav Steinhoff, Robert David, Cornelia A. Lux

**Affiliations:** ^1^Reference and Translation Center for Cardiac Stem Cell Therapy, University of Rostock, 18057 Rostock, Germany; ^2^RTC, BMFZ, Schillingallee 68, 18057 Rostock, Germany

## Abstract

Both stem cell chemokine stromal cell-derived factor-1*α* (SDF-1*α*) and extracellular nucleotides such as adenosine triphosphate (ATP) are increased in ischemic myocardium. Since ATP has been reported to influence cell migration, we analysed the migratory response of bone marrow cells towards a combination of SDF-1 and ATP. Total nucleated cells (BM-TNCs) were isolated from bone marrow of cardiac surgery patients. Migration assays were performed* in vitro*. Subsequently, migrated cells were subjected to multicolor flow cytometric analysis of CD133, CD34, CD117, CD184, CD309, and CD14 expression. BM-TNCs migrated significantly towards a combination of SDF-1 and ATP. The proportions of CD34+ cells as well as subpopulations coexpressing multiple stem cell markers were selectively enhanced after migration towards SDF-1 or SDF-1 + ATP. After spontaneous migration, significantly fewer stem cells and CD184+ cells were detected. Direct incubation with SDF-1 led to a reduction of CD184+ but not stem cell marker-positive cells, while incubation with ATP significantly increased CD14+ percentage. In summary, we found that while a combination of SDF-1 and ATP elicited strong migration of BM-TNCs* in vitro*, only SDF-1 was responsible for selective attraction of hematopoietic stem cells. Meanwhile, spontaneous migration of stem cells was lower compared to BM-TNCs or monocytes.

## 1. Introduction

The contribution of bone marrow cells to cardiac regeneration has been shown by Asahara et al. [1], and, to enhance the efficacy of this physiological mechanism, transplantation of bone marrow cells has since been performed in multiple experimental and clinical studies [2–7]. As the retention of transplanted cells in the myocardium is limited [8] and targeting is crucial for therapeutical effect, approaches to understand and manipulate homing of cells towards sites of injury are of high importance. Among the known factors recruiting bone marrow cells to the heart, SDF-1, a chemokine ligand of the G protein coupled receptor CD184 (CXCR-4), is the most prominent. SDF-1 guides the homing of circulating hematopoietic stem cells towards their bone marrow niches [9]. As SDF-1 is regulated by hypoxia inducible factor-1*α* and thus depends on oxygen tension, it is overexpressed in hypoxic tissues [10, 11]. SDF-1 was demonstrated to be upregulated in rat hearts as early as one hour after induction of ischemia by LAD ligation [12]. After a myocardial infarction, the level of SDF-1 is increased sevenfold [13]. Abbott et al. demonstrated that SDF-1 upregulation after myocardial infarction is necessary for cardiac recruitment of bone marrow cells in a mouse model [14].

However, additional factors with a putative influence on cellular migration are present in injured myocardium. Besides growth factors such as vascular endothelial growth factor (VEGF), interleukin- (IL-) 1 and IL-6, tumor necrosis factor-*α* (TNF-*α*), and complement factors, which are present in infarcted tissue [15, 16], extracellular nucleotides are increased under hypoxic conditions. Adenosine triphosphate (ATP) has been shown to be released in isolated human hearts [17] as well as in cardiomyocytes [18] in response to ischemia. Similarly, in a rat model of cardiac ischemia, a release of uridine triphosphate (UTP) into the circulation was observed [19].

Nucleotides have been implied to play a crucial role in spontaneous migration, if not chemotaxis, of multiple cell types [20]. Neutrophil spontaneous migration is enhanced by ATP, which serves as an autocrine amplifier of chemotactic signals for the cells [21]. Human umbilical cord endothelial cells migrate towards ATP as well as UTP* in vitro* [22]. Rossi et al. demonstrated that nucleotides induce migration of isolated hematopoietic stem cells [23]. In light of these findings, induction or amplification of bone marrow cell motility by nucleotides seems likely.

Many progenitor cell populations as well as differentiated cells have been invoked as regenerative cell populations in bone marrow. To date, a major proportion of clinical trials have been conducted with nucleated bone marrow cells (BM-TNCs) [24]. Which of the multiple cell populations contained in BM-TNCs migrate towards the infarcted myocardium in humans remains to be clarified. In the present study, we performed* in vitro* migration analyses of a BM-TNC product destined for clinical therapy, with a focus on the contained hematopoietic stem cell populations. SDF-1, ATP, and a combination thereof were employed as migratory stimuli.

## 2. Materials and Methods

Bone marrow aspirates were collected from informed donors who gave written consent to the use of their aspirates for research according to the Declaration of Helsinki. The study was approved by University of Rostock Ethical Committee (registered as number A 2010 23) as of April 29, 2010. Bone marrow was aspirated from the sternum immediately before median sternotomy and heparinized (250 i.E./mL).

### 2.1. BM-TNC Isolation

BM-TNCs were processed using the Res-Q 60 BMC System (Thermo Genesis Corp.) according to the manufacturer's instructions. In brief, bone marrow was filtered (200 *μ*m) and transferred to a tube containing a floating chamber. The tube was centrifuged, resulting in density-specific cell selection in the chamber, from where cells were gathered via sterile tubing in a syringe.

### 2.2. Characterization of Freshly Prepared BM-TNC

Cellular composition of starting material BM and cellular product BM-TNCs were analyzed by hemogram (measurement performed by the Institute of Clinical Chemistry and Laboratory Medicine (ILAB), University of Rostock, on a Sysmex XE 5000 machine, Sysmex GmbH, Germany).

### 2.3. *In Vitro* Migration Assay (Modified Boyden Chamber)

Stock solutions of reagents were prepared as follows: adenosine triphosphate disodium salt (ATP, Sigma-Aldrich) was dissolved at a concentration of 4.5 mg/mL in phosphate buffer (DPBS w/o calcium and magnesium, PAN-Biotech GmbH, Germany), sterile-filtered, and stored at −80°C. Recombinant human stromal cell-derived factor-1 (SDF-1, PAN-Biotech) was dissolved in PBS containing 0.1% bovine serum albumin (BSA, Sigma-Aldrich Co., USA) at a concentration of 100 *μ*g/mL under sterile conditions and kept at −20°C. Furthermore, 24 mm transwell inserts (3 *μ*m pore size, Corning Inc., USA) were coated with 10 ng/mL fibronectin (Human Plasma Fibronectin, Merck Millipore, USA) in PBS for 1 hour at room temperature (RT). After washing the inserts three times with PBS, 1 × 10^6^ BM-TNCs per well were seeded into them in 1.5 mL RPMI (PAN-Biotech) containing 1% BSA. Bottom chambers were filled with 2.6 mL of RPMI 1% BSA for evaluation of spontaneous migration or with corresponding amounts of medium containing 100 ng/mL SDF-1, 100 *μ*M ATP, or both. Cells were cultivated in the migration chambers or in 6-well culture plates (incubation control, incubation with SDF-1/SDF-1 + ATP) for 24 h at 37°C 5% CO_2_. To collect migrated cells, inserts were taken out of the migration chambers and cells in the bottom wells were resuspended, transferred to tubes, and washed with PBS. Cells were spun down (300 g, 10 minutes, RT) and cell pellets were resuspended in 50 *μ*L cold PBS containing 2 mM EDTA (Sigma-Aldrich) and 0.5% BSA. FcR blocking reagent (Miltenyi Biotec GmbH, Germany) and antibody staining cocktail (Table 1) were added to the samples, which were then incubated at 4°C for 10 minutes. For intracellular staining, cells were first incubated with near IR live dead stain alone (4°C for 10 minutes) and then fixed with 2% paraformaldehyde (Sigma-Aldrich) in PBS (4°C for 20 minutes), washed with PBS 2 mM EDTA 0.5% BSA, and permeabilized with 0.1% saponin (from Quillaja, Sigma-Aldrich) in PBS 2 mM EDTA 0.5% BSA for 15 minutes at 4°C. Staining of permeabilized cells was continued as described except for near IR live dead stain, using buffers containing 0.1% saponin. Samples were protected from light throughout all the remaining steps. Erythrocyte lysis solution (150 mM ammonium chloride, NH_4_Cl, 10 mM potassium hydrogen carbonate KHCO_3_, and 100 *μ*M ethylenediaminetetraacetic acid EDTA, all from Sigma-Aldrich, were dissolved in distilled water; pH was adjusted to 7.27 and stored at 4°C) was added to the samples, followed by incubation for 10 minutes on ice. Cells were centrifuged for 8 minutes at 4°C and resuspended in PBS/2 mM EDTA/0.5% BSA, an aliquot of the cell suspension was mixed with 3% acetic acid/methylene blue (Stemcell Technologies Inc., Canada), and nucleated cells were counted using a hemocytometer (Carl Roth GmbH + Co. KG, Germany). Remaining samples were then resuspended with PBS and analyzed by flow cytometer.

**Figure 1:**
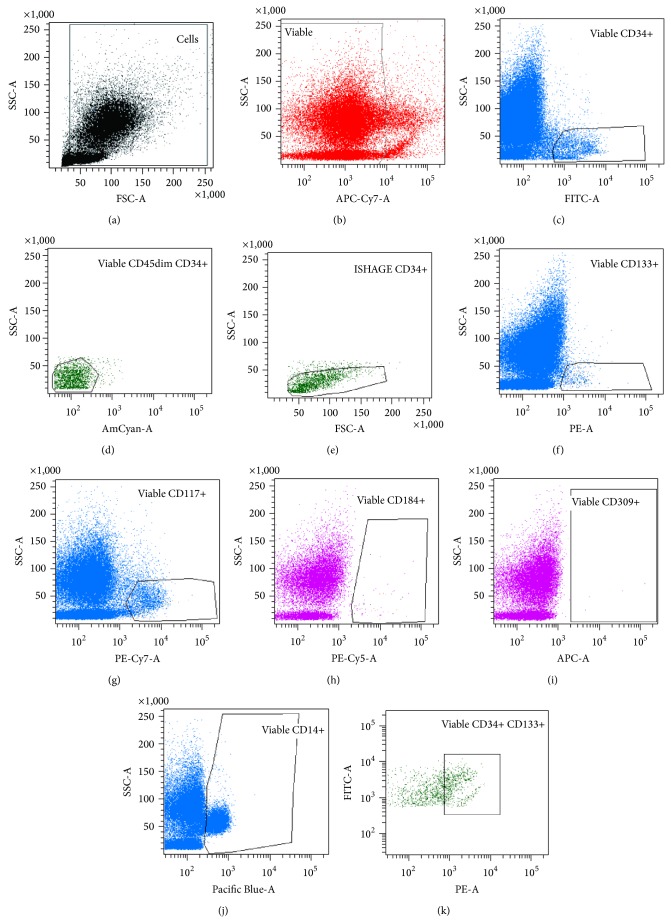
Flow cytometric gating strategy. Debris was excluded in a forward/side scatter (FSC/SSC) dot plot (a); viable cells were defined as cells with low/negative fluorescence in the APC-Cy7 channel (b). Hematopoietic stem cells were selected with an adapted ISHAGE gating strategy [25], exemplified for CD34 in (c)–(e): cells positive for CD34-FITC were gated (c); lymphocytes were excluded based on their high expression of CD45 (d). A FSC/SSC backgate was employed to select CD34+ cells with blast morphology (e). CD133+ and CD117+ HSCs were analysed accordingly ((f) and (g), resp.). CD184+ (CXCR4+), CD309+ (KDR+), and CD14+ cells were gated on viable cells ((h), (i), and (j), resp.). CD34+ CD133+ double positive cell population was gated in a FITC/PE plot (k). Subpopulations coexpressing further markers were selected from the ISHAGE-gated stem cell populations. CD34+ CD133+ CD117+ cells were analysed from ISHAGE-gated CD34+ CD133+ cells, and CD34+ CD117+ cells were analysed based on ISHAGE CD34+ cells.

FACS analysis was performed on BD FACS LSR-II (BD). Single stain samples for compensation were prepared using ArB and ArC bead kit (Life Technologies) according to manufacturer's instructions, as listed in Table 1. Bead single stainings were measured and automated calculation of compensation was performed using FACSDiva software (version 6.1.2, BD). Gating was established with fluorescence-minus-one (FMO) controls. The Boolean gating strategy employed for evaluation of stem cell subpopulations is depicted in Figure 1.

### 2.4. Statistics

Data was evaluated using SigmaPlot (Systat Software Inc., USA). One-way ANOVA was performed for normally distributed variables; the method of Holm-Sidak was employed for post hoc testing. Nonnormally distributed continuous variables as well as variables with unequal variance were evaluated by Kruskal-Wallis one-way analysis of variance of ranks; multiple comparisons versus control group were performed as post hoc analyses according to Dunn's method. Data are presented as mean ± SEM.

## 3. Results and Discussion

### 3.1. Donor Population

As all donors were cardiac surgery patients requiring sternotomy, samples included in the present study represent material of an aged patient population with considerable morbidity, typically presenting arterial hypertension and hypercholesterolemia. Therefore, conclusions drawn from our analyses may not be directly transferable to cell products derived from young, healthy donors. In contrast, the donor population is fairly representative for potential recipients of cell products for cardiac regeneration. While the donor population was heterogeneous in terms of age, donor gender was predominantly male (Table 2).

### 3.2. Composition of Freshly Isolated BM-TNC

BM-TNC isolated by Res-Q 60 BMC System included all mature hematopoietic cell populations as well as stem and progenitor cells. Flow cytometric analysis of fresh BM-TNC is included in Figure 3.

Stem and progenitor cell populations were defined by their markers CD117, CD34, and CD133 as well as SDF-1 receptor CD184 (CXCR4). CD14, expressed by monocytes, served as a control marker for mature cells. CD14 was expressed on 4.03 ± 0.37% of viable cells, a value within the expected range, as compared to monocyte concentrations described in the literature (6.3 ± 3.3% of leukocytes in bone marrow of healthy donors [26], 4.10% of MNC in donor bone marrow grafts [27]). A minor fraction of cells expressed CD184 (14.56 ± 2.23% of viable cells), much less than that reported by Dotsenko et al. [28]. CD309 positive cells were very infrequent and could not be analysed reliably. CD309 was therefore excluded from marker evaluation. The most frequent stem cell population was CD117+ (1.16 ± 0.09% of viable cells), followed by CD34+ as the most common marker in flow cytometric stem cell analysis (0.85 ± 0.07% of viable cells). In comparison to previous studies, we found lower overall numbers of stem cells [26, 28–32]. Several factors may account for this discrepancy: the individual bone marrow source may influence stem cell frequencies, as sternal bone marrow aspirates may be more diluted with peripheral blood than the iliac crest aspirates used by Tendera et al. [30] or Theilgaard-Mönch et al. [29]; Dor et al. [32] found 5.1% CD117+ and 3.6% CD34+ cells in human bone marrow derived from a single deceased human donor. Age and morbidity of the donor population may influence stem cell number, although this may not explain the difference to the study of Dotsenko et al. [28], which was comparable to our own study in terms of donor collective as well as bone marrow source. Differences in cell isolation procedure may influence the proportions of specific cell types; the gating strategy we employ is more restrictive than Dotsenko et al.'s due to multiple backgates and, as a result, may lead to lower stem cell numbers. In all multicolor flow cytometry analyses, spreading of populations occurs even in properly compensated samples and may lead to underestimation of target populations [33]. It is plausible that spreading may be more pronounced in the 8-fold staining we employed than in the commonly used 4-fold ISHAGE staining.

Stem cell populations overlapped considerably (Table 3). Again, our findings differed from Tendera et al. as well as Dotsenko et al.; notably, CD184+ cell proportion in CD34+ cells was considerably lower in our study compared to these and other studies [34–36].

### 3.3. Migration of BM-TNC

**Figure 2:**
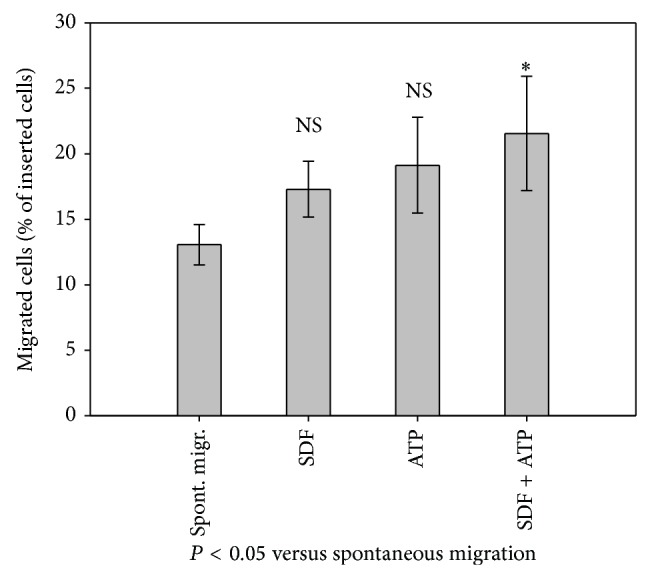
BM-TNCs migrated significantly towards SDF-1 + ATP. BM-TNCs were subjected to 100 ng/mL SDF-1 and/or 100 *μ*M ATP as migratory stimulant in a Boyden chamber for 24 h; medium was employed to estimate spontaneous migration in absence of any migratory stimuli. Migrated BM-TNCs were retrieved from the lower chamber and counted in a hemocytometer, *n* ≥ 4.

Total BM-TNC migrated significantly only towards a combination of SDF-1 and ATP, although a trend of migration towards either SDF-1 or ATP alone was detectable (Figure 2). This is a discrepancy compared to the findings of Seeger et al., who reported a 50% increase of BM-TNC invasion by 100 ng/mL SDF-1 [37]. However, assay parameters differed notably between Seeger's and our experiments (cell isolation procedure, larger pore size, and Matrigel coating of Boyden chamber). Yet, the positive impact of ATP on SDF-1-triggered migration is in accord with the work of Ratajczak et al. [38].

**Figure 3:**
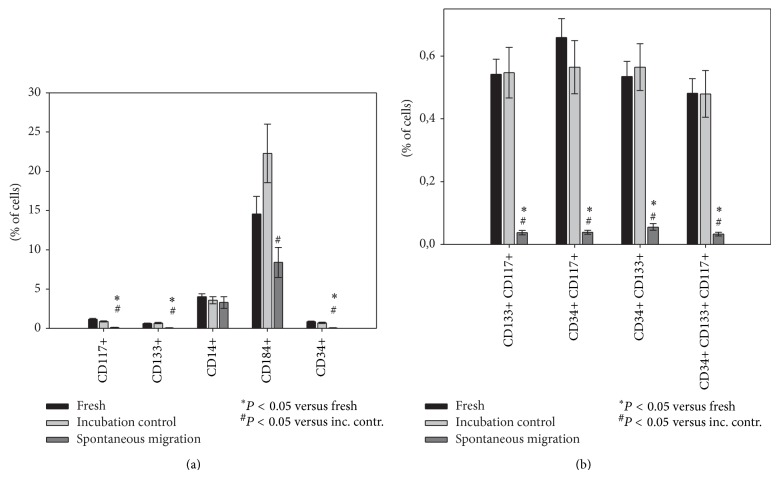
Stem cell populations were diminished significantly after spontaneous migration. Multicolor flow cytometric analyses were performed on BM-TNC after isolation (“fresh”), after cultivation (“incubation control”), or on BM-TNC retrieved from lower Boyden chamber after cultivation for 24 h (“spontaneous migration”). Cell populations (a) and stem cell subpopulations (b) were evaluated as shown in Figure 1. CD34+, CD133+, and CD117+ cells formed significantly lower percentages of BM-TNC which had undergone spontaneous migration (a). The effect was even more pronounced for populations expressing multiple stem cell markers (b). Incubation had no influence on CD34+, CD133+, and CD117+ cells or on their subpopulations but led to an increase in CD184+ cells, *n* = 10.

As a sizable number of cells migrated spontaneously in the absence of any migratory stimulus, that is, via spontaneous migration, we performed flow cytometric analysis of these cells and compared their marker expression profile to those of freshly isolated BM-TNC as well as BM-TNC cultivated in assay medium (“incubation control”) (Figure 3). Whereas cultivation for 24 hours did not affect expression of any of the examined markers significantly, the spontaneous migration group displayed an unanticipated change in the expression pattern. All included stem cell populations as well as subpopulations were diminished compared to both fresh and cultivated BM-TNCs. The CD184+ proportion was reduced significantly in cells after spontaneous migration as compared to cultivated BM-TNC. The fraction of CD184+ cells tended to increase with cultivation; however, this trend was not significant, possibly due to the large variability of expression for this marker (Figure 3(a)).

These findings may be explained by two separate effects:

1. stem cell motility might be lower than motility of mature cells in the absence of specific stimuli;
2. spontaneous migration and/or transmigration through a porous membrane might cause a reduction in stem cell marker expression (e.g., by internalization). Therefore, migrated stem cells would no longer be detected as such by flow cytometric analysis.

For CD117, CD34, and CD133, internalization in response to specific triggers has been described [39–41]. The triggers, however, vary conspicuously, and no reports of internalization of these markers through migration have yet been published.

**Figure 4:**
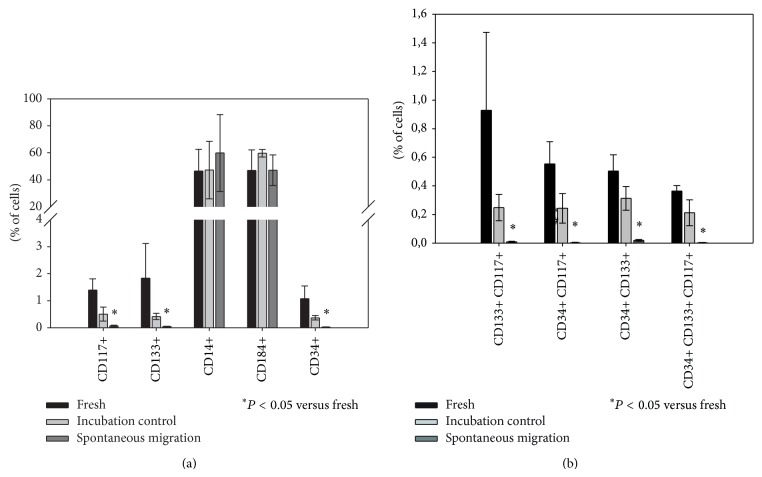
Diminished stem cell populations after spontaneous migration may not be explained by marker internalization. Intracellular multicolor flow cytometric analyses were performed on BM-TNC after isolation (“fresh”), after cultivation (“incubation control”), or on BM-TNC retrieved from lower Boyden chamber after cultivation for 24 h (“spontaneous migration”). Cell populations (a) and stem cell subpopulations (b) were evaluated as shown in Figure 1. After spontaneous migration, CD34+, CD133+, and CD117+ cells (a) as well as populations expressing multiple stem cell markers (b) formed lower percentages of BM-TNC, *n* = 3.

In order to clarify the relevant mechanism for our finding, we performed intracellular staining after incubation and spontaneous migration. Again, stem cell markers were detected on or in fewer cells after spontaneous migration, suggesting that migrated stem cells were not concealed by marker internalization in our previous experiment (Figure 4). A level of significance was only reached for spontaneously migrated cells in comparison to fresh BM-TNC; however, a clear trend towards fewer stem cells was displayed compared to the incubation control.

Therefore, lower motility of stem cells seems the most probable explanation of our results. In fact, Shin et al. found that, due to their lower lamin A : B stoichiometry, stem/progenitor cells possess more rigid nuclei than mature blood cells, which hinders their capacity to deform and migrate through 3 *μ*m pores [42]. This accords well with our present data and may account for the low numbers of stem cells in the spontaneous migration fraction.

**Figure 5:**
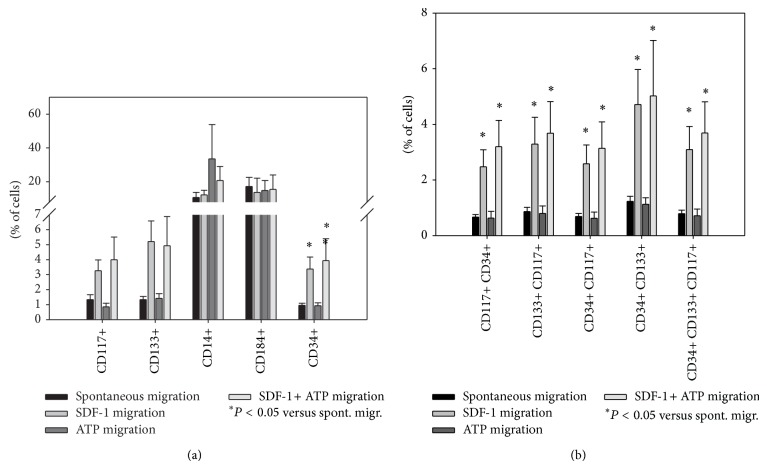
CD34+ stem cells as well as subpopulations positive for multiple stem cell markers migrated significantly towards SDF-1 + ATP. After* in vitro *migration for 24 h towards medium (“spontaneous migration”), 100 ng/mL SDF-1 (“SDF-1 migration”), 100 *μ*M ATP (“ATP migration”), or a combination of both (“SDF-1 + ATP migration”), BM-TNCs were analysed by flow cytometry. Cell populations (a) and stem cell subpopulations (b) were evaluated as described in Figure 1. CD34+ cell number was significantly elevated in BM-TNCs after migration towards SDF-1 or SDF-1 + ATP (a); subpopulations expressing multiple stem cell markers also displayed higher concentrations in SDF-1 or SDF-1 + ATP migrated fraction (b), *n* ≥ 4.

Presence of SDF-1 or ATP altered the composition of migrated cells substantially (Figure 5). After migration along an SDF-1 or a combined SDF-1 and ATP gradient, CD34+ stem cells were present in significantly higher proportion. A significant increase was detected by ANOVA for CD117+ and CD133+ cell percentages as well; however, post hoc analysis did not confirm this. Subpopulations expressing multiple stem cell markers were significantly augmented. In contrast, monocytes remained unaffected. Cell composition after migration towards ATP corresponded to that of the spontaneous migration fraction.

**Figure 6:**
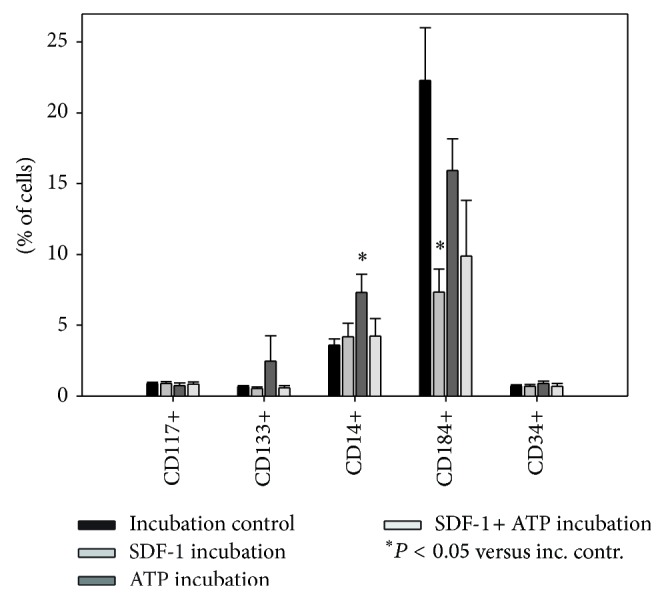
The proportion of CD184+ cells was lower after incubation with SDF-1. Following incubation for 24 h with medium (“incubation control”), 100 ng/mL SDF-1 (“SDF-1 incubation”), 100 *μ*M ATP (“ATP incubation”), or a combination of both (“SDF-1 + ATP incubation”), BM-TNCs were analysed by flow cytometry. Cell populations were evaluated as described in Figure 1. CD184+ cell proportion decreased significantly after incubation with SDF-1, while other populations were not affected. CD14+ cell population was increased after incubation with ATP, *n* ≥ 4.

Despite the fact that CD184 is the receptor for SDF-1 and plays a fundamental role in stem cell homing along SDF-1 gradients, no increase in CD184+ cells in the SDF-1 or SDF-1 + ATP-migrated fractions was detected. As Signoret et al. demonstrated, CD184 is internalized after binding of its ligand [43]. To verify whether receptor internalization took place during our experiment, we analyzed cells incubated with SDF-1/SDF-1 + ATP (Figure 6). A significant decrease in CD184 expression was indeed observed after incubation with SDF-1. Incubation with SDF-1 + ATP led to a noticeable, albeit not quite significant, decrease as well. More CD14+ cells were detected after incubation with ATP. A possible explanation for this finding could be ATP-triggered secretion of IL-1*β*, a well-known inducer of CD14 [44, 45]. No significant changes occurred in the other cell populations.

## 4. Conclusion

The present work gives an account of the migration potential of cell populations contained in a bone marrow-derived cell product. The migratory stimuli employed, namely, stem cell chemokine SDF-1 and free nucleotide ATP, are both present in myocardial tissue after ischemic injury. To our knowledge, we are the first to give a comprehensive analysis of specific stem cell populations after* in vitro *migration towards these stimuli.

We found that total BM-TNCs migrated significantly towards SDF-1 and ATP, while migration towards SDF-1 alone did not reach the level of significance. It is conceivable that ATP may contribute to BM-TNC homing after transplantation. Differentiated hematopoietic cells as well as progenitors have been shown to express P2 nucleotide receptors [46] which may detect ATP and trigger diverse cellular reactions such as migration [47]. Considering plausible locations for ATP release in injured myocardium as well as cell populations migrating significantly towards ATP, however, ATP-triggered migration might not be responsible for regenerative effects of BM-TNC.

In cardiac regeneration, hibernating tissue regions with lowered metabolism despite continuing viability seem to profit most from cell transplantation [48]. Whereas ATP is set free to the intracellular space by necrotic cell death [49], total tissue ATP is decreased in hibernating myocardium [50, 51] and disruptive cardiomyocyte death is not typical for these areas. Considering ATP to be a migratory stimulus along with SDF-1, targeting of transplanted cells and physiological recruiting of cells directly from bone marrow might lead to cell presence in injured tissue with suboptimal susceptibility to be rescued.

Our analysis of cell populations migrating towards SDF-1, ATP, or a combination of both showed that CD34+ stem cells as well as subpopulations expressing multiple stem cell markers displayed significant migration towards SDF-1 + ATP. The percentage of these populations, however, was also increased significantly in cells migrated towards SDF-1 alone. The selective attraction of stem cell populations therefore relies on SDF-1, not on ATP. The increase of total migrating BM-TNC may be accounted for by neutrophils, which form the majority of leukocytes and are known to react to nucleotides as migratory stimuli [21]. Whereas neither the most active cell populations nor their exact mechanism of action has been completely clarified to date, it seems improbable that proinflammatory, short-living differentiated cells such as neutrophils would produce a beneficial effect in hibernating cardiac tissue.

CD184+ cell proportion was not changed by migration towards SDF-1, although CD184 is its main receptor. However, receptor downregulation may account for this finding, confirmed by the CD184+ cell decrease we detected after incubation with SDF-1.

Stem cells displayed lower spontaneous migration than differentiated cells as shown by extra- and intracellular marker analysis; this finding corresponds to the report of Shin et al. that stem cell nuclei are more rigid and less likely to be deformed, thereby hindering cellular migration through pores. Whereas the extent of stem cell transmigration may thus be limited, specificity of stem cell recruitment into injured tissue is likely enhanced.

Our findings emphasize the complexity of cell migration in presence of different stimuli. Populations displaying elevated migratory potential, such as cells coexpressing multiple stem cell markers, may be especially promising for therapy or as biomarkers. Whether these populations are also highly active by paracrine secretion or differentiation or via stimulation of cardiomyocytes and resident cardiac stem cells remains to be tested. Culture expansion or* in vitro* generation of these rare populations may serve to provide cells for regenerative targeting in the future. Application of ATP in addition to SDF-1, on the other hand, did not enhance stem cell-specific migration and its clinical use is not recommendable based on our present work. Ultimately, the reduced spontaneous migration of stem cells compared to differentiated cells may be made use of innovative methods for gentle, label-free stem cell purification.

## Figures and Tables

**Table 1:** Antibody cocktail for multicolor staining (flow cytometry).

Antibody	Fluorochrome	Manufacturer
CD34	FITC	Miltenyi Biotec
CD133	PE	Miltenyi Biotec
CD184	PE-Cy5	Becton Dickinson (BD), USA
CD117	PE-Cy7	BD
CD309	APC	Miltenyi Biotec
CD14	V450 (Pacific Blue)	BD
CD45	V500 (AmCyan)	BD
Live dead stain	Near IR (detection channel: APC-Cy7)	Life Technologies Corp., USA

**Table 2:** Bone marrow donor characteristics.

Donor number	26
Proportion of male donors	88.5%
Mean donor age	64.2 (range 40–85) y

**Table 3:** Stem cell population overlap in BM-TNC.

Subpopulation	% of CD34+ cells	% of CD133+ cells	% of CD117+ cells	% of CD34+ CD133+ cells
CD34+ CD133+	62.32 ± 2.39	84.79 ± 2.91	—	—
CD34+ CD133+ CD117+	55.64 ± 2.43	75.69 ± 3.11	41.21 ± 2.60	89.04 ± 1.47
CD34+ CD117+	77.29 ± 1.87	—	56.80 ± 3.11	—
CD133+ CD117+	—	86.08 ± 1.72	46.89 ± 2.79	—
CD34+ CD184+	24.31 ± 3.02	—	—	—
CD117+ CD184+	—	—	20.13 ± 2.68	—
CD34+ CD133+ CD184+	11.65 ± 1.81	16.59 ± 2.77	—	19.39 ± 3.06
